# Cost-effectiveness of rezvilutamide *versus* bicalutamide and androgen-deprivation therapy in patients with highvolume, metastatic, hormone-sensitive prostate cancer

**DOI:** 10.3389/fphar.2023.1269129

**Published:** 2024-01-09

**Authors:** Huina Wu, Lei Sun, Rui Feng, Huiyue Zhang, Ke Tang, Shuo Wang, Jing Nie

**Affiliations:** ^1^ Department of Pharmacy, Shandong Second Provincial General Hospital, Jinan, Shandong, China; ^2^ College of Pharmacy, Linyi University, Linyi, Shandong, China; ^3^ School of Business Administration, Shandong Women’s University, Jinan, Shandong, China

**Keywords:** cost-effectiveness, rezvilutamide, bicalutamide, highvolume mHSPC, partitioned survival model

## Abstract

**Background:** Rezvilutamide, a novel androgen-receptor inhibitor with limited blood-brain barrier penetration, exhibits significant antitumour activity against highvolume, metastatic, hormone-sensitive prostate cancer (mHSPC). In this study, we aimed to compare the cost-effectiveness of rezvilutamide and bicalutamide as first-line treatments for untreated prostate cancer among Chinese patients, in order to evaluate the efficacy of rezvilutamide.

**Methods:** In this study, we utilized partition survival model to assess the cost-effectiveness of rezvilutamide and bicalutamide treatments for highvolume mHSPC. The model was developed using TreeAge Pro 2022 software and relied on clinical data obtained from the CHART trial. Transition probabilities were estimated from the reported survival probabilities in trials using parametric survival modeling. From the perspective of the Chinese healthcare system, we calculated quality-adjusted life years (QALYs), incremental cost-effectiveness ratio (ICER), and lifetime cost. A lifetime horizon and an annual discount rate of 5% were employed. To address modeling uncertainties, we conducted one-way sensitivity analysis and probabilistic sensitivity analysis.

**Results:** The cost of rezvilutamide *versus* bicalutamide were $62700 and $13200. Rezvilutamide had an ICER of $41900 per additional QALYs gained compared with bicalutamide. Research indicated that rezvilutamide achieved at least an 28.20% probability of cost-effectiveness at the threshold of $38223.34/QALY. One-way sensitivity analysis revealed that the results were sensitive to utility of PD. Scenario analysis showed that rezvilutamide was cost-effectiveness if its price was reduced by more than 10%.

**Conclusion:** Based on the analysis at the current price, rezvilutamide was found to be less cost-effective for patients with highvolume mHSPC compared to bicalutamide in China.

## 1 Introduction

Prostate cancer (PC) stands as the most prevalent malignant tumor within the male genitourinary system. According to the World Health Organization (WHO) GLOBOCAN statistics in 2020, its incidence rate ranks second among all male malignant tumors, just behind lung cancer (Sung et al., 2021). In China, prostate cancer’s incidence and mortality contribute to 8.2% and 13.6% of the global figures, respectively. The incidence and mortality rate of prostate cancer in China consistently rank among the highest, displaying an upward trend year by year (Wei et al., 2021). This escalating burden has bestowed significant psychological, economic, and caregiving pressures upon both patients and caregivers, steadily emerging as the primary health challenge affecting men’s wellbeing in China. As such, addressing the multifaceted impact of prostate cancer on Chinese men’s health has become an imperative area of concern for healthcare practitioners and policymakers alike.

The conventional therapeutic approach for metastatic hormone sensitive prostate cancer centers aroud androgen deprivation therapy (ADT). However, recent advances in the field have unveiled the immense potential of combination docetaxel with ADT (a treatment modality known as Doc-ADT) This combination therapy has demonstrated remarkable superiority over ADT alone, gaining recognition as a recommended treatment option for patients diagnosed with mHSPC. Additionally, the introduction of second-generation androgen receptor (AR) antagonists has revolutionized the management of this disease. These agents effectively delay the onset of castration resistance and contribute to extended overall survival in patients. Notably, novel androgen receptor inhibitors like apalumide and enzarumide have emerged, showcasing improved clinical outcomes for patients while offering the advantage of lower toxicity (Armstrong et al., 2019; Chi et al., 2019; Davis et al., 2019). Despite these encouraging advancements, it is essential to remain vigilant about potential adverse effects associated with AR antagonists such as enzalutamide and apalutamide. Reports have highlighted their potential risks, including epilepsy, fatigue, and rash. As the medical community continues to explore and optimize treatment strategies for mHSPC, careful consideration of the benefits and risks of these therapies remains paramount to ensure the wellbeing of patients.

Rezvilutamide presents a novel androgen receptor inhibitor boasting independent intellectual property rights in China. The drug has made significant advancements and enhancements in its molecular structure, rendering it more suitable for the Chinese prostate cancer population. Through a randomized, open-label phase 3 clinical trial, the combination of rezvilutamide with ADT was compared to the combination of bicalutamide with ADT in patients afficted with metastatic and hormone sensitive prostate cancer. The research findings remarkable outcomes for the rezvilutamide group. A 56% reduction in the risk of imaging progression or death was observed compared to the bicalutamide group. Furthermore, patients in the rezvilutamide group experienced significantly prolonged survival time, with a 42% reduction in the risk of death; Remarkable benefits were also observed in terms of secondary and exploratory efficacy endpoints for the rezvilutamide group. Notably, the administration of revalutamide was well-tolerated, exhibiting excellent safety profiles (Gu et al., 2022).

Although the preceding experiments have demonstrated promising efficacy of combining rezvilutamide with castration therapy in the treatment of high tumor burden metastatic hormone sensitive prostate cancer, the current literature has not yielded any pharmacoeconomic research on this specific treatment regimen, both domesticall and internationally. Hence, this study aims to bridge this knowledge gap by employing a partitioned survival model from the perspective of China’s health system to investigate the economic advantages of rezvilutamide combined with castration therapy as compared to bicalutamide combined with castration therapy for managing high tumor burden metastatic hormone sensitive prostate cancer. The primary objective is to offer valuable insights and reference for clinical treatment selection and national health decision-making processes.

## 2 Methods

### 2.1 Model structure

**FIGURE 1:**
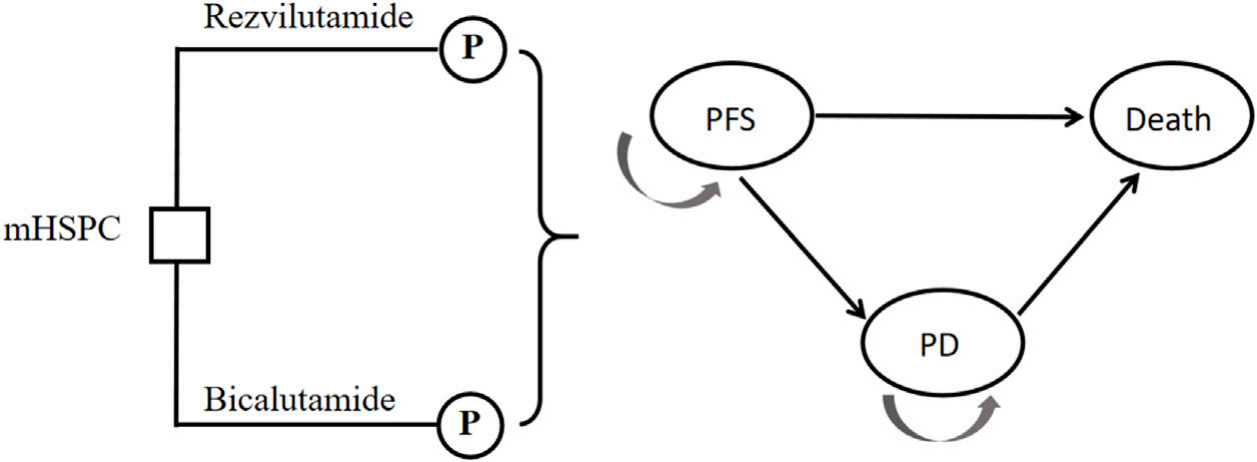
Model diagram for patients with mHSPC. PFS, progression-free survival; PD, progressive disease.

This study adopts a frontier approach in pharmacology, focusing on China’s health system. It endeavors to establish a partitioned survival model, commonly employed in pharmacoeconomic research on advanced cancer, to simulate the progressive disease (PD) status of patients with large volume, metastatic, and hormone sensitive prostate cancer. For the purpose of modeling, Treeage 2022 software is employed to construct partitioned survival model comprising three states: Progression-Free Survival (PFS), Progressive Disease (PD), and death. Estimate the proportion of patients who survived *t* cycles using the area under the OS curve, and estimate the proportion of patients who survived and PFD using the area under the PFS curve. Additionally, estimate the proportion of surviving patients and PD patients based on the difference between OS and PFS curves. This model is built upon the clinical development trajectory of the disease, as illustrated in Figure 1. The probabilities of transitioning between states are derived through survival analysis. Several key assumptions are considered within this model. Firstly, it assumes an initial cohort of 1,000 patients entering the model with a state of PFS and subsequently commencing medication. the endpoint of treatment is set as either PD or death. Secondly, the incidence of adverse reactions during each treatment cycle is assumed to align with the findings from the CHART trial, However, potential adjustments to drug dosage and treatment modifications due to adverse reactions are not accounted for in this study. Additionally, adverse reactions occurring after changing the treatment method following PD are not considered in the model. Thirdly, it is assumed that once a patient transitions to PD status, the current treatment regimen should be discontinued, and the treatment plan consistent with the CHART trial should be adopted. Lastly, all patients included in the study are assumed to be inpatients.

The OS data in this study were sourced from the NCT03520478 trial (Gu et al., 2022). To obtain PFS and OS time sample data for the cohort, PFS and OS curves of the NCT03520478 test were carefully analyzed using GetData Graph Digitizer software. The individual patient data (IPD) were obtained using the RStudio software following the approach outlined in the literature (Ramamurthy et al., 2019; Nie et al., 2023). Subsequently, the fitted K-M survival curve was generated using Stata 14 software, allowing for an observation of how well it aligned with the original survival curve. To assess the appropriateness of the chosen fitting function, the new PFS and OS curves were simulated using the Weibull distribution parameters. A comparison was made between the median PFS time and median OS time derived from the original survival curve. This comparison helped judge the rationality of the selected fitting function. In order to further validate the reconstructed curves, they were visually simulated following the guidelines from the literature (Gu et al., 2022). Additionally, the Akaike Information criterion (AIC) and Bayesian Information criterion (BIC). were employed for thorough examination (Table 1). Based on this rigorous analysis, it was concluded that the lognormal distribution provides the best fit for PFS and OS data concerning bicalutamide and rezvilutamide (Table 2).

**TABLE 1:** AIC and BIC of the fit function.

Bicalutamide		Exponential	Gamma	Gompertz	Weibull	Loglogistic	Lognormal
PFS	AIC	1,116.78	1,091.10	1,114.96	1,097.67	1,084.56	1,074.12
	BIC	1,120.57	1,098.68	1,122.54	1,105.25	1,092.15	1,081.71
OS	AIC	1,281.77	1,261.13	1,274.51	1,263.65	1,259.72	1,255.66
	BIC	1,285.56	1,268.72	1,282.09	1,271.23	1,267.31	1,263.24
Rezvilutamide		exponential	gamma	gompertz	weibull	loglogistic	lognormal
PFS	AIC	853.64	847.54	854.13	848.80	846.49	841.47
	BIC	857.43	855.11	861.71	856.38	854.06	849.05
OS	AIC	931.65	922.00	930.96	923.66	921.10	916.11
	BIC	935.44	929.58	938.53	931.23	928.67	923.68

**TABLE 2:** Distribution parameters of lognormal.

	Bicalutamide				Rezvilutamide			
	PFS		OS		PFS		OS	
μ	3.03		3.74		3.90		4.27	
σ	0.95		1.05		1.27		1.20	

In 2020, the average life expectancy in China was recorded as 77.93 years (National Health Commission, 2022). Given that the median age of patients in the CHART study is 69.2 years, a time duration of 10 years was chosen to encompass the entirety of the patient’s expected lifespan. Each cycle in the model corresponds to the 28-day treatment period observed in the clinical trial. The key outcomes assessed in the model include quality-adjusted life years (QALYs) associated with the treatment scheme and incremental cost-effectiveness ratios (ICERs). To adhere to the Guidelines for China Pharmaceutical Economics Evaluation 2020 (Ge, 2020), an annual discount rate of 5% was applied to both costs and utility in this study. Sensitivity analysis was conducted, considering a range of discount rates from 0% to 8%. The simulation time limit of the model was set at 10 years, with each cycle lasting 28 days (based on the medication period). Aligning with the recommendations of the World Health Organization (World Health Organization, WHO), the willingness to pay (willingness to pay, WTP) threshold was determined to be 1 to 3 times *per capita* GDP (gross domestic product, GDP). Therefore, for this study, the WTP threshold (Eichler et al., 2004) was established at 3 times the *per capita* GDP in 2022, amounting to 36581.3911 dollary (12193.79*3 = 36581.3911 doller) (National Buildings Organisation Statistics, 2022).

### 2.2 Costs and utility values

The evaluation is approach from the perspective of the Chinese healthcare system, and as such, it considers only direct medical expenses. These expenses encompass various elements, such as drug costs for rezvilutamide and bicalutamide, follow up monitoring expenses, best supportive care (BSC) costs, terminal care cost (TCC), and the management of serious adverse effects (SAEs). To, derive accurate cost data, the unit price of rezvilutamide and bicalutamide in China were obtained from the Shandong drug centralized procurement platforms. For other cost data, relevant literature was referenced. In order to reflect the most recent economic, all the costs were adjusted for inflation, converting them into 2022 US dollars, by taking into account the Chinese Consumer Price Index (CPI) and the 2022 exchange rate (6.7261 RMB/US dollar). For calculating the drug cost of chemotherapy, a base-case body surface area (BSA) of 1.72 m^2^ was assumed. Detailed information on all these costs can be found in Table 3 to calculate the drug cost of chemotherapy. The utility preferences of PFS and PD states related to mHSPC were 0.76 and 0.68, which were derived from in a CHART study (Ramamurthy et al., 2019). All information is listed in Table 3. Please refer to Supplementary Material S1 for information on cycle-dependent probabilities of progression and death. Additionally, to better present our research results, we have included a ‘Cheers’ report checklist in Supplementary Material S2.

**TABLE 3:** Model parameters: baseline values, ranges, and distributions for sensitivity analysis.

Parameter	Expected value	Range	Distribution	Source
Drug costs ($)				
Rezvilutamide/cycle	880.45	704.36–1,056.54	fixed	Shandong drug centralized procurement platforms (2022)
bicalutamide/cycle	104.56	83.65–125.47	fixed	Shandong drug centralized procurement platforms (2022)
AEs costs ($)				
Hypertension	12.15	9.72–14.58	gamma	Zhang et al. (2021)
Hypertriglyceridaemia	14.90	11.92–17.88	gamma	Zhao et al. (2019)
Follow up monitoring cost ($)				
CT scan	211.56	169.25–253.87	gamma	Zhang et al. (2021)
PSA tests	25.00	20.00–30.00	gamma	Zhang et al. (2021)
Diagnostic costs	67.2	53.76–80.64	gamma	local charge
12-lead electrocardiogram	56.20	44.96–67.44	gamma	local charge
Laboratory tests	34.49	27.59–41.39	gamma	local charge
Terminal care cost	2104.25	1,683.41–2525.10	gamma	Ming et al. (2023)
Utility				
PFS	0.76	0.608–0.912	beta	Ramamurthy et al. (2019)
PD	0.68	0.544–0.816	beta	Ramamurthy et al. (2019)
Probabilities, %				
Rezvilutamide				
Hypertension	8.00%		beta	Gu et al. (2022)
Increased weight	6.00%		beta	Gu et al. (2022)
Hypertriglyceridaemia	7.00%		beta	Gu et al. (2022)
bicalutamide				
Hypertension	7.00%		beta	Gu et al. (2022)
Increased weight	4.00%		beta	Gu et al. (2022)
Hypertriglyceridaemia	2.00%		beta	Gu et al. (2022)
Discount (%)	0.05	0–8	beta	

## 3 Results

### 3.1 Base-case analysis

As shown in Table 4, the cumulative cost for rezvilutamide and bicalutamide were observed to be$62700 and $13200, respectively. This comparison indicates that the rezvilutamide group incurred an additional cost of $49500 but also achieved a gain 1.18 quality-adjusted life years (QALYs)over the bicalutamide group. Employing the Incremental Cost-Effectiveness Ratios (ICERs) to assess the cost-effectiveness of the two treatment approaches, the resulting ICER value was $41900, surpassing the specified WTP threshold.

**TABLE 4:** Base results of rezvilutamide ***versus*** bicalutamide.

Strategy	Cost	Incr cost	Eff	Incr Eff	ICER	NMB
bicalutamide	13200		3.52			12100
rezvilutamide	62700	49500	4.70	1.18	41900	11700

### 3.2 Sensitivity analysis

**FIGURE 2:**
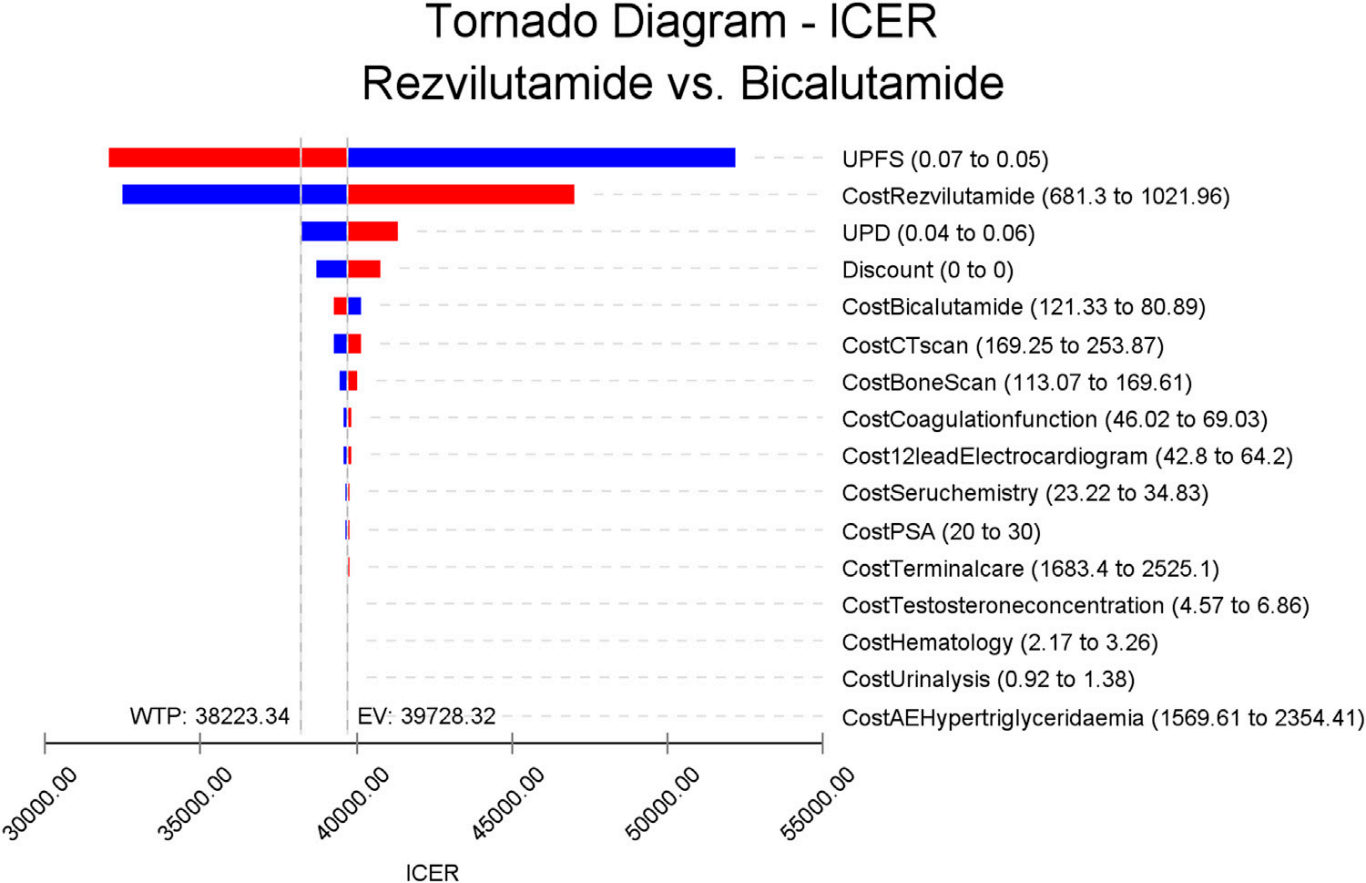
Tornado diagram of the one-way deterministic sensitivity analysis (bicalutamide *versus* rezvilutamide). PSA, prostate-specific antigen; CT, computed tomography; PD, progressed disease; PFS, progression-free survival. Red: High value, Blue: Low value.

The tornado diagram depicted in Figure 2 illustrates the sensitivity analysis of the Cost-Effectiveness Ratios (ICERs) for bicalutamide *versus* rezvilutamide. Among the factors considered, the ICERs were found to be most sensitive to the utility of progression-free survival (PFS), followed by the cost of rezvilutamide and the utility of progressive disease (PD). The insightful analysis suggests that potential improvements in the utility of PFS and a decrease in the price of rezvilutamide could render rezvilutamide a more cost-effective option compared to bicalutamide. In fact, significant advancements in the utility of PFS and price reductions for rezvilutamide might even position it as the most effective solution. Furthermore, the study demonstrated that when other parameters varied within their upper and lower limits during sensitivity analysis, the research results consistently aligned with the findings from the basic case analysis. This observation indicates that the conclusions drawn from basic case analysis are generally stable and robust.

**FIGURE 3:**
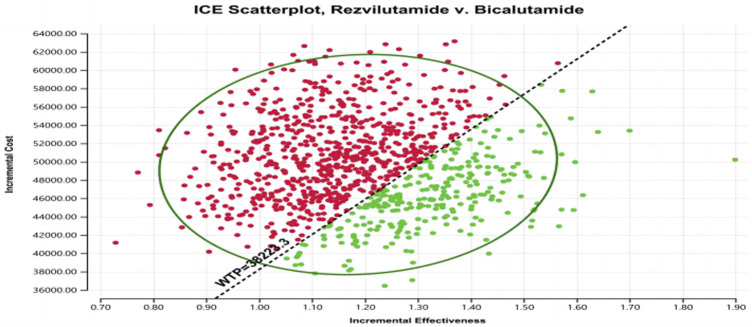
Scatter plot of probability sensitivity analysis. WTP, willingness to pay. Red: ICER value greater than WTP. Green: ICER value less than WTP.

**FIGURE 4:**
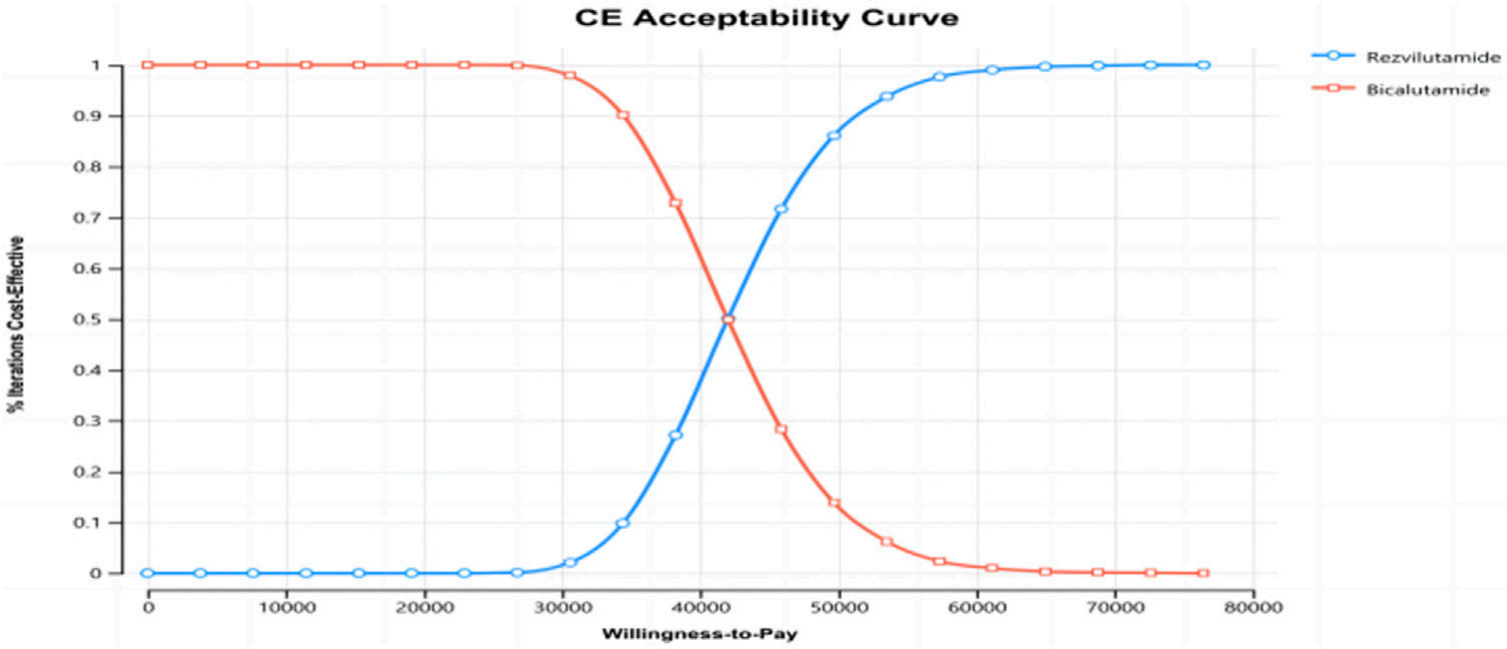
Cost-effectiveness acceptable curve (CEAC).

In the context of the probabilistic sensitivity analysis, the outcomes suggest that, at a specific willingness-to-pay (WTP) level of $38,223.3 per quality-adjusted life year (QALY), rezvilutamide exhibits a 28.2% likelihood of being considered cost-effective when compared to bicalutamide (as depicted in Figure 3 and Figure 4). As a result, the present evidence suggests that, for patients, bicalutamide currently represents the more cost-effective for patients.

### 3.3 Scenario analysis

The Scenario analysis reveals that with a 10% reduction in the price of rezvilutamide, its ICER amounts to 38013.75, which falls below the WTP threshold. Consequently, rezvilutamide demonstrates economic viability under these circumstances.

## 4 Discussion

In recent years, China has witnessed a notable rise incidence rate and mortality of prostate cancer, primarily attributed to factors such as population aging, the adoption of western dietary patterns, and the widespread implementation of prostate specific antigen (PSA) screening. These trends have placed patients at a considerable risk of disease progression and mortality, underscoring the urgent need for novel, effective, and safe drug treatments to improve their prognosis. In the past, treatment options for mHSPC were limited. However, the introduction of second-generation Androgen receptor (AR) inhibitors brought a ray of hope to mHSPC therapy. Despite this positive development, most of these second-generation AR inhibitors were imported medications, and the registered clinical studies primarily focused on Western populations. Consequently, there exists a significant dearth of evidence-based medicine specifically tailored to meet the needs of Chinese patients in clinical practice. This highlights the crucial requirement for research and innovation in the field of prostate cancer treatment in China.

The clinical efficacy of rezvilutamide has garnered widespread recognition within the academic community. Notably, in the esteemed"Chinese Society of Clinical Oncology (CSCO) Prostate Cancer Diagnosis and Treatment Guidelines (2022 Edition)" (Chinese Society of Clinical Oncology, 2022), the combined use of rezvilutamide and ADT for managing high tumor burden mHSPC has received a Level I recommendation (Class 1A evidence). This acknowledgment underscores the therapeutic significance of rezvilutamide in addressing this challenging medical condition. Moreover, on 28 June 2022, rezvilutamide received approved for listing from the National Medical Products Administration, and remarkably, it attained inclusion in the medical insurance coverage within a mere 7 months from its 3listing approval. This rapid process the country’s dedication to reforming the medical insurance system and prioritizing the wellbeing of its people by alleviating medical burdens. Additionally, it serves as a testament to the nation’s profound recognition and support for pioneering pharmaceutical enterprises, encouraging further advancements in the field of innovative drug development.

Rezvilutamide stands as a pioneering second-generation AR inhibitor that has been independently developed within the confines of China. The Phase III clinical trial data has unveiled its remarkable potential in treating high tumor burden mHSPC patients, exemplifying a substantial extension in overall survival (OS) and imaging progression free survival (rPFS, validated through an independent film review). Notably, the utilization of rezvilutamide led to a remarkable42% reduction in the risk of mortality and an impressive54% decrease in disease progression. Moreover, the paramount, aspect of this groundreaking drug lies in its exceptional safety profile. Throughout the trial, a mere 0.9% of patients necessitated permanent discontinuation, while only2.5% required a dosage reduction due to adverse reactions. These figures are notably lower compared to the historical data of similar pharmaceuticals, accentuating the favorable safety and tolerability of rezvilutamide (Gu et al., 2022).

From a perspective firmly rooted within China’s healthcare system, this investigation expertly employed partitioned survival model to assess the economic efficacy of rezvilutamide when compared to bicalutamide for treating high volume, metastatic, and homone sensitive prostate cancer. The findings revealed that the ICER of the rezvilutamide group stood at $41900/QALY, contrasting significantly with the WTP threshold of $38223.3/QALY, based on three times China’s *per capita* GDP in 2022 (Xie et al., 2022). Under these circumstances, the feasibility of the rezvilutamide group appears economically constrained. Nonetheless, the Scenario analysis casts a ray of hope. By envisioning a 10% reduction in rezvilutamide’s price, the ICER plummets to $38013.75, thus dipping below the WTP threshold. At such a juncture, rezvilutamide proves its economic value. Notably, the results from the single factor sensitivity analysis spotlight the PFS state utility value, rezvilutamide price, PD utility value and Bank rate as having substantial influences on the model’s outcomes.

This study, however, has its fair share of limitations that necessitate acknowledged: (1) The Pharmacoeconomics evaluation is grounded in the CHART study, which, unfortunately, boasts a limited follow-up time. Consequently, the utilization of survival extrapolation through the parameter method to derive long-term survival data introduces an element of uncertainty into the study findings. (2) It is important to recognize that this study did not take into consideration other potential posterior treatment options. This omission may result in discrepancies between the obtained results and the actual outcomes. (3) The study solely accounted for the top two serious adverse reactions related to the treatment while neglecting other potential adverse reactions. As a consequence, there may exist a certain degree of bias between the calculated cost and utility values and the real-world scenario. (4) The differences between the true incremental survival and QALYs and the predictions from the probabilistic sensitivity analysis approach will depend on many factors. Although there is difference the degree of difference between predicted and actual results is expected to be less significant as data maturity increases, the overestimation or underestimation of true incremental life years and QALYs by the PSM is difficult to predict in any specific situation.

## 5 Conclusion

In spite of the considerable advancements in PFS observed in patients with highvolume mHSPC, our study indicates that rezvilutamide is unlikely to be cost-effective for the majority of adults when compared to bicalutamide, considering a WTP threshold of $38223.3/QALY from the perspective of the Chinese healthcare system. Nevertheless, a promising economic advantage can be achieved if rezvilutamide is incorporated into the NRDL (National Reimbursement Drug List) and undergoes a 10% reduction in price.

## Data Availability

The original contributions presented in the study are included in the article/Supplementary Material, further inquiries can be directed to the corresponding author.
